# Principles of Human Physiology, with Their Chief Applications to Psychology, Pathology, Therapeutics, Hygiene, and Forensic Medicine

**Published:** 1853-06

**Authors:** 


*Principles of Human Physiology, with their chief applications to Psychology, Pathology, Therapeutics, Hygiene, and Forensic Medicine.* By Wm. B. Carpenter, M. D., F. R. S., F. G. S., etc., etc. Fifth American from the fourth and enlarged London edition. With three hundred illustrations. Edited, with additions, by Francis Gurney Smith, M. D., Professor of the Institutes of Medicine in the Medical Department of Pennsylvania College, etc. Philadelphia: Blanchard & Lea. 1853. 8vo., pp. 1091.

Our readers would not thank us for commending to them Dr. Carpenter's works on Physiology. They need not be informed that for several years past these works have been emphatically *the* English text books in this department of medical knowledge. In this volume the author has entirely remodeled the preceding edition. This has been rendered necessary by the important modifications of physiological science which have, within a very few years past, changed very considerably its aspect. The work, in fact, is a new treatise on the principles of human physiology in their various scientific and practical relations. It is an exponent of the present condition of this rapidly progressive science. This is the highest recommendation which a work of such a character can receive. Its importance to the medical practitioner, as well as the medical pupil, will be appreciated in proportion as the true connection between physiology and pathology is correctly uuderstood. On this point the remarks of the author in his introduction, appear to us eminently just. It is undoubtedly true that, (to quote his language,) "the phenomena of disease have been isolated from those of health, as if they belonged to quite a distinct category, and were dependent on a set of causes altogether dissimilar. It has been too much lost sight of that every diseased action is but a perversion by *excess*, by *diminution*, or by *depravation*, of some natural function; and that only through an acquaintance with the latter can the former be understood, (otherwise than in a merely empirical fashion,) either as to its cause, its nature, or its tendencies." It is also, as it seems to us, in accordance with the philosophy of medicine, that "just where our physiological knowledge is most precise, and its generalizations most comprehensive, our pathological information is most advanced, and most fruitful in practical results."

